# Optimal Triage for COVID-19 Patients Under Limited Health Care Resources With a Parsimonious Machine Learning Prediction Model and Threshold Optimization Using Discrete-Event Simulation: Development Study

**DOI:** 10.2196/32726

**Published:** 2021-11-02

**Authors:** Jeongmin Kim, Hakyung Lim, Jae-Hyeon Ahn, Kyoung Hwa Lee, Kwang Suk Lee, Kyo Chul Koo

**Affiliations:** 1 College of Business Korea Advanced Institute of Science and Technology Seoul Republic of Korea; 2 Division of Infectious Disease Department of Internal Medicine Yonsei University College of Medicine Seoul Republic of Korea; 3 Department of Urology Yonsei University College of Medicine Seoul Republic of Korea

**Keywords:** COVID-19, decision support techniques, machine learning, prediction, triage

## Abstract

**Background:**

The COVID-19 pandemic has placed an unprecedented burden on health care systems.

**Objective:**

We aimed to effectively triage COVID-19 patients within situations of limited data availability and explore optimal thresholds to minimize mortality rates while maintaining health care system capacity.

**Methods:**

A nationwide sample of 5601 patients confirmed with COVID-19 until April 2020 was retrospectively reviewed. Extreme gradient boosting (XGBoost) and logistic regression analysis were used to develop prediction models for the maximum clinical severity during hospitalization, classified according to the World Health Organization Ordinal Scale for Clinical Improvement (OSCI). The recursive feature elimination technique was used to evaluate the maintenance of model performance when clinical and laboratory variables were eliminated. Using populations based on hypothetical patient influx scenarios, discrete-event simulation was performed to find an optimal threshold within limited resource environments that minimizes mortality rates.

**Results:**

The cross-validated area under the receiver operating characteristic curve (AUROC) of the baseline XGBoost model that utilized all 37 variables was 0.965 for OSCI ≥6. Compared to the baseline model’s performance, the AUROC of the feature-eliminated model that utilized 17 variables was maintained at 0.963 with statistical insignificance. Optimal thresholds were found to minimize mortality rates in a hypothetical patient influx scenario. The benefit of utilizing an optimal triage threshold was clear, reducing mortality up to 18.1%, compared with the conventional Youden index.

**Conclusions:**

Our adaptive triage model and its threshold optimization capability revealed that COVID-19 management can be achieved via the cooperation of both the medical and health care management sectors for maximum treatment efficacy. The model is available online for clinical implementation.

## Introduction

The high incidences of infection, critical illness, and mortality due to COVID-19 have placed unprecedented burdens on international health care systems. In response, the World Health Organization (WHO) guidelines have recommended that all countries prepare for infection surges in their health care facilities and implement appropriate triage protocols [1]. Unfortunately, these guidelines fail to provide a one-size-fits-all approach that works for individual regions while accounting for unique outbreak surges.

Numerous prognostic models have been developed to ensure effective triage for COVID-19 patients [2-7]. While these models exhibit modest predictive accuracy, their generalizability has been questioned due to their confinement to single clinical outcome measures and reductions in their discrimination performance when using insufficient data. Most importantly, the classification thresholds of these prediction models, which are crucial for ensuring effective resource utilization by health care systems, have been neglected, thereby limiting their practicality. To overcome these models’ shortcomings, combing multi-institutional data with advanced prediction models, such as those using machine learning and simulation modeling, is needed.

COVID-19 is associated with significant disruptions to most health care infrastructures. Therefore, an adjustable risk stratification model that considers the resource availability of various regions, as well as one that identifies patients who will likely require hospitalization and intensive care, will help to reduce these systems’ burdens. In this study, we propose an adaptive triage model that takes into account deficits in established health care resources due to the COVID-19 pandemic. Our study has several main contributions. The first contribution is a powerful and interpretable prediction model using extreme gradient boosting (XGBoost) and Shapley additive explanations (SHAP) that provides accurate prognoses to facilitate preemptive treatments, thereby ensuring improvements in patient survival outcomes. The second contribution is the ability to apply the model with readily available assessment parameters using the recursive feature elimination (RFE) technique, thereby maintaining its reliability in data-limited environments [8,9]. The third contribution is the consideration of resource availability at either the facility or national level relative to varying patient influx volumes by employing the discrete-event simulation (DES) technique.

Our study objectives were 3-fold. First, we sought to develop a baseline prediction model with an explanatory feature for triaging COVID-19 patients. Second, based on this model, we aimed to utilize the RFE technique to develop feature-eliminated models that would help ensure efficient resource utilization under limited data availability. Finally, we set out to develop an adaptive triage model using the DES technique to assist in efficient resource utilization under limited health care resources.

## Methods

### Ethics Statement

This study was approved by an institutional ethics committee (2020-0883-001) and the Korea Disease Control and Prevention Agency (KDCA) epidemiological survey and analysis committee (20201120_4a). All study procedures complied with the 1946 Declaration of Helsinki and its 2008 update.

### Patient Cohort

We retrospectively retrieved the demographic, clinical, laboratory, and disease outcome records of 5628 patients who were confirmed with SARS‐CoV‐2 by real-time reverse transcription-polymerase chain reaction using nasopharyngeal/oropharyngeal swab or sputum specimens until April 2020. The data were collected and comprehensively managed by the KDCA. Among 10,774 patients consecutively diagnosed with COVID-19 within this time frame, data on 52.2% (5628/10,774) of the patient population were publicized for research purposes after excluding patients with any missing data. The database did not account for the location of diagnosis within Korea. The database included patients who had been treated and released from quarantine or hospitalization, as well as those who died from COVID-19 sequelae. The criteria for patient release included obtaining 2 consecutive negative results at least 24 hours apart and an asymptomatic status. Among the 5628 patients, 27 patients with missing clinical severity data were excluded, resulting in a final development cohort of 5601 patients.

### Covariates and Outcome Definitions

Baseline data collected at each patient’s diagnosis were used for model development. Demographic data included patient age, sex, systolic and diastolic blood pressure, heart rate, body temperature, and BMI. Medical comorbidities included hypertension, diabetes mellitus, heart failure, cardiovascular disease, asthma, chronic kidney disease, chronic obstructive pulmonary disease, chronic liver disease, autoimmune disease, dementia, malignancy, and pregnancy. Clinical findings included a history of fever (temperature ≥37.5℃), cough, sputum production, myalgia, fatigue, sore throat, rhinorrhea, dyspnea, vomiting, nausea, diarrhea, headache, and altered consciousness. Laboratory data included hemoglobin, hematocrit, white blood cell count, %leukocyte, and platelet count. Each patient’s maximum clinical severity during quarantine or hospitalization was classified according to the WHO Ordinal Scale for Clinical Improvement (OSCI) [10].

### Statistical Analysis

#### Model Development

Multivariate logistic regression (LR) and XGBoost were used to select the best performing prediction model using all available clinical and laboratory data [11]. The models were developed and cross-validated using data from 5037 (89.9%) patients and were then revalidated using a hold-out cohort of 564 (10.1%) patients. Performance metrics were calculated using 10-fold cross-validation to avoid any overfitting. Model development was performed using the *caret* package in R Statistical Package (version 4.0.5; R Project for Statistical Computing). The best performing model derived from XGBoost was defined as Model 1 and was used as a baseline model for RFE.

#### Variable Elimination

The RFE technique was used to evaluate the extent of the maintenance of model performance when various predictors were eliminated. RFE was performed for the following 2 models that incorporated all clinical data with and without laboratory data: Model 1 (clinical data with laboratory data) and Model 2 (clinical data without laboratory data). SHAP was used to rank each variable based on its significance to the models for its desirable properties, including local accuracy, missingness, and consistency [12]. At each RFE iteration, the lowest-ranked feature was eliminated, the model was refitted, and its performance was assessed using 10-fold cross-validation. The feature-eliminated models (Model 3: limited clinical data with laboratory data and Model 4: limited clinical data without laboratory data) were then selected at a point wherein the number of features was minimized while differences in area under the receiver operating characteristic curve (AUROC) values remained statistically insignificant. The 4 classification models were revalidated with the hold-out cohort to avoid any overfitting. Analysis was performed using *caret* and the *SHAPforXGBOOST* package in R.

#### Model Interpretation and Comparison

To interpret Model 1, we used SHAP as it provides visible post-hoc interpretability to black-box machine learning models [12]. Patient-specific plots were created by aggregating the SHAP score of each variable for a specific prediction.

The hyperparameters of the XGBoost algorithm were optimized to maximize its AUROC values using a simple grid search with 10-fold cross-validation. Accuracy, AUROC, sensitivity, positive predictive value (PPV), and negative predictive value (NPV) were calculated at 90% specificity using the *pROC* package in R. CIs of the performance measures were then calculated using a stratified bootstrap method with 2000 replicates.

#### Threshold Optimization

##### DES and Patient Influx Generation

The DES technique replicates complex behaviors and interactions among individuals, populations, and their environments. Therefore, it has been widely used to form more effective clinical decisions to minimize mortality rates under medical resource constraints [13]. Thus, we applied DES to identify the optimal threshold within limited medical resource environments that minimizes mortality rates, as calculated by *n* (*total deaths*) / *n* (*total patients*), using the *simmer* R package.

First, we ran a simulation using different COVID-19 historical epidemic patient influx scenarios (H1, H2, H3, and H4) that were observed between February 2020 and February 2021 (Multimedia Appendix 1) [14]. Second, hypothetical patient influx scenarios were created using the susceptible-infectious-recovered (SIR) model for disease spread [15]. The total population calculated was fixed at 60,000, considering that the largest historical influx observed in South Korea was H4 (58,654 cumulative patients). We defined initial conditions at time t=0, S(0), I(0), and R(0), and I(0) and R(0) were fixed at 6 and 0, respectively. The recovery rate gamma was set at 0.05 because the average COVID-19 recovery time was 20.1 days [16]. The transmission rate beta ranged between 0.75 and 5 when generating influxes with different R0 (basic reproduction rate) levels. The number of newly confirmed patients per day was obtained from the SIR modeling data (Multimedia Appendix 2).

##### Probability Generation

Out-of-fold prediction results of the 10-fold cross-validation were aggregated to generate an empirical probability distribution of the disease severity probability. We used the results of Model 3 because of its high performance and its potential use in instances of limited diagnostic tools. Inverse transformation sampling was performed on the empirical probability distribution function, which was approximated using Gaussian kernel density estimation and linear interpolation [17]. The process was performed separately for severe and nonsevere patients, with sampled probabilities being randomly matched with generated patient influx rates while maintaining the prevalence of severe patients. The prediction probability distribution of the out-of-fold samples and the generated prediction probability distribution are presented in Multimedia Appendix 3.

##### Simulation Scenarios

Patients with a severe disease probability above the threshold are directed to the intensive care unit (ICU), with admission to this unit then being dependent on its current capacity. Rejected patients are directed to the general ward along with those who have a severe disease probability below the threshold. The probability of severe disease patients dying while in the ICU was 0.507, while it was 0.990 for those outside of the ICU [18]. We assumed that nonsevere patients would survive regardless of ICU admission. Patient deaths were categorized as follows: resource-independent deaths, wherein severe patients died despite ICU care (type I); resource-dependent deaths, wherein severe patients died due to ICU unavailability (type II); and threshold-dependent deaths, wherein severe patients died after being incorrectly classified as “nonsevere” and subsequently directed to the general ward (type III).

The maximum capacity of the ICU was established as 504 beds based on the number of isolation beds under negative pressure [14]. To estimate the distribution of length of stay, we used a previously suggested gamma distribution with a shape parameter of 1.5488 and a rate parameter of 0.1331 for those who died, and with a shape parameter of 0.8904 and a rate parameter of 0.0477 for those who survived to approximate the median and IQR [18,19]. Simulations were repeated 20 times for each influx scenario to ensure robustness.

## Results

### Patient Characteristics

Descriptive characteristics of the training and hold-out cohorts are provided in Tables 1 and 2. A total of 5330 (95.2%) patients exhibited nonsevere disease symptoms with an OSCI value <6, while 271 (4.8%) exhibited severe disease symptoms with an OSCI value ≥6.

**Table 1:** Demographic characteristics.

Variable		Total cohort (N=5601)		Training cohort (N=5037)			Hold-out cohort (N=564)			*P* value^a^
		Value, n (%) or mean (SD)	Missing data, %	Value, n (%) or mean (SD)	Missing data, %	Value, n (%) or mean (SD)		Missing data, %		
**Age (years)**			0.0%		0.0%			0.0%	.41	
	0-9	66 (1.2%)		61 (1.2%)		5 (0.9%)				
	10-19	205 (3.7%)		185 (3.7%)		20 (3.6%)				
	20-29	1110 (19.8%)		988 (19.6%)		122 (21.6%)				
	30-39	564 (10.1%)		513 (10.2%)		51 (9.0%)				
	40-49	739 (13.2%)		652 (12.9%)		87 (15.4%)				
	50-59	1141 (20.4%)		1039 (20.6%)		102 (18.1%)				
	60-69	907 (16.2%)		809 (16.1%)		98 (17.4%)				
	70-79	545 (9.7%)		495 (9.8%)		50 (8.9%)				
	≥80	324 (5.8%)		295 (5.9%)		29 (5.1%)				
Sex (male)		2310 (41.2%)	0.0%	2073 (41.2%)	0.0%	237 (42.0%)		0.0%	.73	
**BMI (kg/m^2^)**			21.4%		21.5%			20.9%	.65	
	<18.5	259 (4.6%)		236 (4.7%)		23 (4.1%)				
	18.5-22.9	1854 (33.1%)		1666 (33.1%)		188 (33.3%)				
	23.0-24.9	1035 (18.5%)		929 (18.4%)		106 (18.8%)				
	25.0-29.9	1045 (18.7%)		938 (18.6%)		107 (19.0%)				
	≥30	207 (3.7%)		185 (3.7%)		22 (3.9%)				
**Medical history**										
	Diabetes mellitus	688 (12.3%)	0.1%	620 (12.3%)	0.1%	68 (12.1%)		0.0%	.92	
	Hypertension	1198 (21.4%)	0.1%	1087 (21.6%)	0.1%	111 (19.7%)		0.0%	.32	
	Heart failure	59 (1.1%)	0.1%	52 (1.0%)	0.1%	7 (1.2%)		0.0%	.81	
	Cardiovascular disease	179 (3.2%)	0.3%	156 (3.1%)	0.3%	23 (4.1%)		0.4%	.26	
	Asthma	128 (2.3%)	0.1%	118 (2.3%)	0.1%	10 (1.8%)		0.0%	.48	
	Chronic obstructive pulmonary disease	40 (0.7%)	0.1%	38 (0.8%)	0.1%	2 (0.4%)		0.0%	.43	
	Chronic kidney disease	55 (1.0%)	0.1%	48 (1.0%)	0.1%	7 (1.2%)		0.0%	.67	
	Malignancy	145 (2.6%)	0.1%	134 (2.7%)	0.1%	11 (2.0%)		0.0%	.39	
	Chronic liver disease	83 (1.6%)	5.8%	75 (1.6%)	5.7%	8 (1.5%)		6.7%	>.99	
	Autoimmune disease	38 (0.7%)	5.9%	32 (0.7%)	5.8%	6 (1.1%)		6.9%	.37	
	Dementia	224 (4.2%)	5.9%	203 (4.3%)	5.8%	21 (3.7%)		6.7%	.81	

^a^Differences between groups were analyzed using the Welch *t* test for continuous variables, the Mann-Whitney *U* test for ordinal variables, the chi-square test for categorical variables with frequencies above 5, and the Fisher exact test for categorical variables with frequencies below 5. Two-sided *P* values are reported.

**Table 2:** Clinical characteristics.

Variable		Total cohort (N=5601)		Training cohort (N=5037)			Hold-out cohort (N=564)			*P* value^a^
		Value, n (%) or mean (SD)	Missing data, %	Value, n (%) or mean (SD)	Missing data, %	Value, n (%) or mean (SD)		Missing data, %		
**Systolic blood pressure (mmHg)**			2.5%		2.5%			2.7%	.60	
	<120	1306 (23.3%)		1177 (23.4%)		129 (22.9%)				
	120-129	1138 (20.3%)		1012 (20.1%)		126 (22.3%)				
	130-139	1084 (19.4%)		977 (19.4%)		107 (19.0%)				
	140-159	1418 (25.3%)		1281 (25.4%)		137 (24.3%)				
	≥160	513 (9.2%)		463 (9.2%)		50 (8.9%)				
**Diastolic blood pressure (mmHg)**			2.5%		2.5%			2.7%	.04	
	<80	2102 (37.5%)		1878 (37.3%)		224 (39.7%)				
	80-89	1797 (32.1%)		1601 (31.8%)		196 (34.8%)				
	90-99	1056 (18.9%)		971 (19.3%)		85 (15.1%)				
	≥100	504 (9.0%)		460 (9.1%)		44 (7.8%)				
Heart rate (bpm)		85.8 (SD 15.1)	2.3%	85.8 (SD 15.0)	2.3%	86.3 (SD 15.4)		2.5%	.47	
Body temperature (°C)		36.9 (SD 0.6)	0.7%	36.9 (SD 0.6)	0.8%	37.0 (SD 0.6)		0.7%	.86	
**Symptoms**										
	Fever	1302 (23.3%)	0.1%	1168 (23.2%)	0.1%	134 (23.8%)		0.0%	.80	
	Cough	2331 (41.6%)	0.1%	2103 (41.8%)	0.1%	228 (40.4%)		0.0%	.58	
	Sputum	1611 (28.8%)	0.1%	1460 (29.0%)	0.1%	151 (26.8%)		0.0%	.29	
	Sore throat	872 (15.6%)	0.1%	779 (15.5%)	0.1%	93 (16.5%)		0.0%	.57	
	Rhinorrhea	617 (11.0%)	0.1%	560 (11.1%)	0.1%	57 (10.1%)		0.0%	.51	
	Myalgia	920 (16.4%)	0.1%	820 (16.3%)	0.1%	100 (17.7%)		0.0%	.41	
	Fatigue	233 (4.2%)	0.1%	207 (4.1%)	0.1%	26 (4.6%)		0.0%	.65	
	Shortness of breath	665 (11.9%)	0.1%	608 (12.1%)	0.1%	57 (10.1%)		0.0%	.19	
	Headache	963 (17.2%)	0.1%	873 (17.3%)	0.1%	90 (16.0%)		0.0%	.45	
	Altered consciousness	35 (0.6%)	0.1%	31 (0.6%)	0.1%	4 (0.7%)		0.0%	.78	
	Vomiting	244 (4.4%)	0.1%	210 (4.2%)	0.1%	34 (6.0%)		0.0%	.05	
	Diarrhea	516 (9.2%)	0.1%	457 (9.1%)	0.1%	59 (10.5%)		0.0%	.32	
**Laboratory values**										
	Hemoglobin (g/dL)	13.3 (SD 1.8)	27.2%	13.3 (SD 1.8)	26.7%	13.2 (SD 1.8)		31.6%	.41	
	Hematocrit (%)	39.2 (SD 5.0)	27.2%	39.3 (SD 4.9)	26.7%	39.1 (SD 5.2)		31.7%	.56	
	Lymphocyte proportion (%)	29.2 (SD 11.7)	27.6%	29.3 (SD 11.7)	27.1%	28.2 (SD 11.0)		32.1%	.09	
	Platelet count (/μL)	236,697 (SD 82,897)	27.1%	236,776 (SD 82,534)	26.7%	235,943 (SD 86,395)		31.4%	.86	
	White blood cell count (/μL)	6126 (SD 2824)	27.1%	6121 (SD 2841)	26.7%	6167 (SD 2666)		31.4%	.75	
WHO OSCI^b^ ≥6		271 (4.8%)	0.0%	242 (4.8%)	0.0%	29 (5.1%)		0.0%	.80	
Pregnancy		19 (0.3%)	0.4%	17 (0.3%)	0.3%	2 (0.4%)		0.5%	>.99	
Pregnancy weeks		0.05 (SD 1.1)	0.4%	0.06 (SD 1.1)	0.4%	0.03 (SD 0.5)		0.5%	.40	

^a^Differences between groups were analyzed using the Welch *t* test for continuous variables, the Mann-Whitney *U* test for ordinal variables, the chi-square test for categorical variables with frequencies above 5, and the Fisher exact test for categorical variables with frequencies below 5. Two-sided *P* values are reported.

^b^WHO OSCI: World Health Organization Ordinal Scale for Clinical Improvement.

### Model Performance

The cross-validated AUROC values of the XGBoost and LR models were 0.965 (95% CI 0.958-0.972) and 0.938 (95% CI 0.911-0.959), respectively (*P*=.04). We chose the XGBoost model as our baseline Model 1 since it outperformed the LR model across all performance measures. Regarding the AUROC, we also examined XGBoost’s outperformance across 4 different severity endpoints (Multimedia Appendix 4). An online clinical decision-support system based on Model 3 is provided for clinical implementation [20].

### Model Interpretability

**Figure 1:**
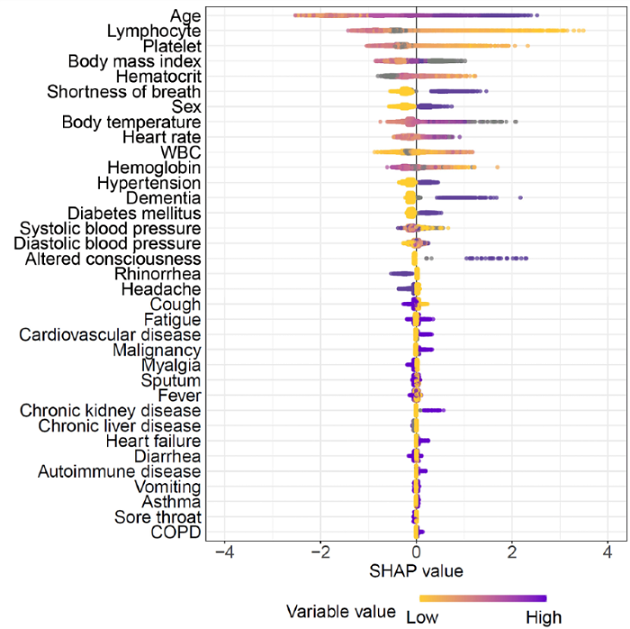
Relationships between each feature and Shapley additive explanations (SHAP) values. Summary plot in which each dot point represents the SHAP value of a patient in the data set used to construct the developed model. The dots are plotted for every feature used to fit the baseline model, excluding 2 features (pregnancy and number of weeks pregnant) that were not selected for the developed model. The SHAP values are displayed in rank order, based on their feature importance, along the y-axis as calculated by averaging the absolute SHAP values of each dot. A point’s location on the x-axis shows its impact on the predictive output of the model. Purple indicates a relatively high feature value, while yellow represents a relatively low feature value. Grey dots represent missing values. COPD: chronic obstructive pulmonary disease; WBC: white blood cell.

**Figure 2:**
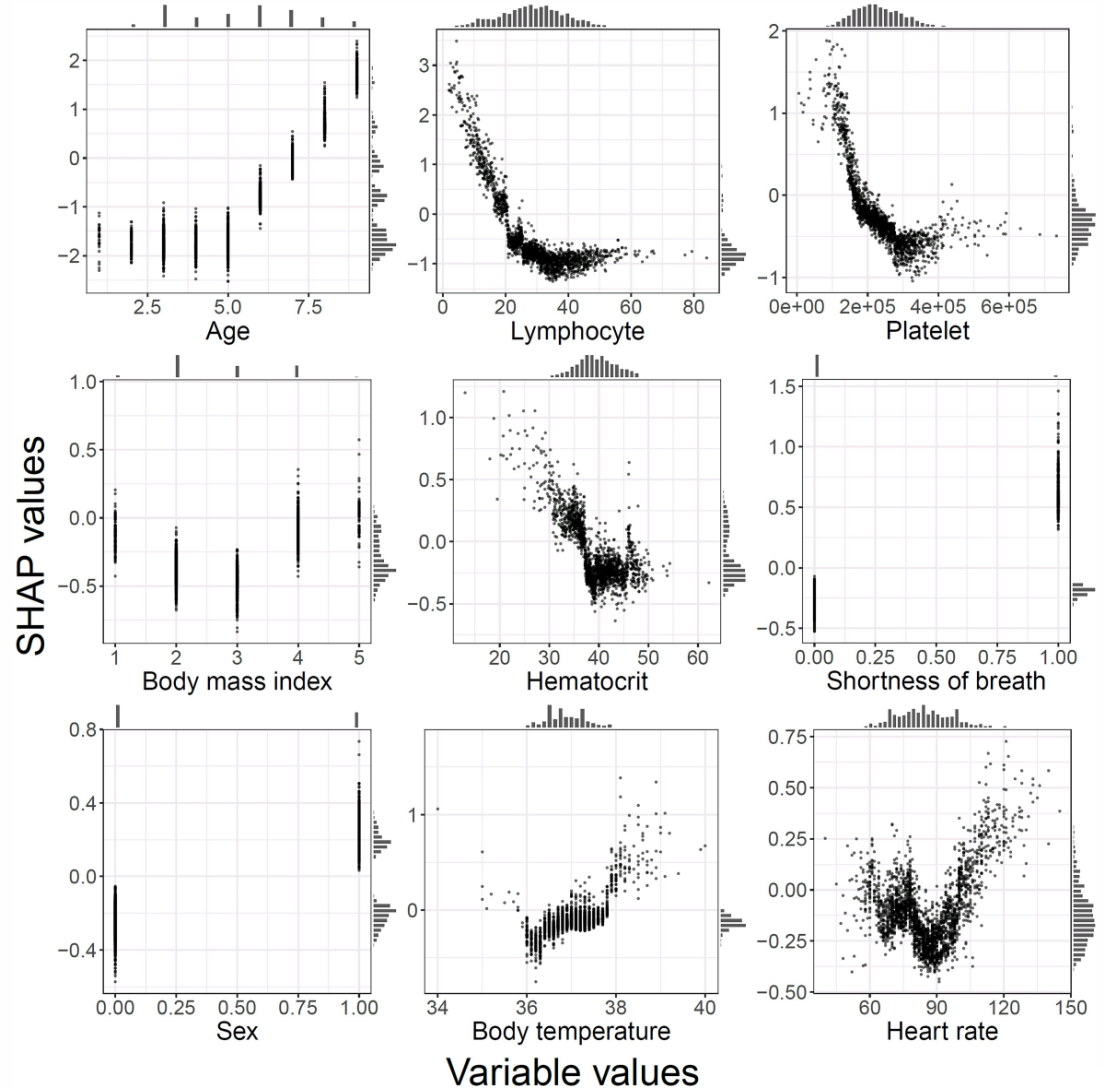
Relationships between each feature and Shapley additive explanations (SHAP) values. Dependence plots for each of the top 9 important features, including patient age, lymphocyte proportion, platelet count, BMI, hematocrit, shortness of breath, sex, body temperature, and heart rate. Each scatter plot shows the impact of each feature on the predictions made by the study model. The x-axis represents the variables’ values, and the y-axis represents their SHAP values. The inflection points indicate the nonlinear impact of a feature on the model’s prediction.

According to SHAP, age and lymphocyte count were the most important risk factors for predicting disease severity of OSCI ≥6 (Figure 1). Patient age, lymphocyte proportion, platelet count, BMI, hematocrit, and heart rate all exhibited nonlinear influences in predicting disease severity (Figure 2). In addition to the overall impact of each feature on the model’s output, SHAP provides patient-specific influences of each variable on the predicted disease severity (Multimedia Appendix 5).

### Predictive Performance Under Limited Data Availability

An AUROC of 0.965 (95% CI 0.958-0.972) was obtained with Model 1, which included all 37 variables. Notably, a reduction in its performance was found to be insignificant when 20 variables were eliminated, resulting in Model 3 (Multimedia Appendix 6 and Multimedia Appendix 7). Model 1 achieved both a sensitivity and specificity greater than 90%. Model 3 achieved a sensitivity of 88% and a PPV of 31% at the specificity level of 90%. Model 3 still outperformed the LR model regarding all performance measures.

An AUROC of 0.946 (95% CI 0.936-0.956) was obtained with Model 2, which included 32 variables. The reduction in performance was found to be insignificant when 21 variables were eliminated, resulting in Model 4 (Multimedia Appendix 7 and Multimedia Appendix 8). Models 2 and 4 achieved sensitivities of 84% and 81%, respectively, at a fixed specificity level of 90% (Table 3). Significant differences in AUROCs were observed when laboratory variables were excluded in these models, which implied that the laboratory variables had a solid discriminative power (all *P*≤.01).

**Table 3:** Comparison of model performance.

Model	Number of variables	AUROC^a^, value (95% CI)	Specificity, value (95% CI)	Sensitivity, value (95% CI)	Accuracy, value (95% CI)	PPV^b^, value (95% CI)	NPV^c^, value (95% CI)
1	37	0.965 (0.958-0.972)	0.900 (0.892-0.909)	0.905 (0.868-0.942)	0.900 (0.892-0.908)	0.314 (0.295-0.335)	0.995 (0.993-0.997)
2	32	0.946 (0.936-0.956)	0.900 (0.891-0.908)	0.839 (0.793-0.884)	0.897 (0.888-0.905)	0.297 (0.276-0.319)	0.991 (0.988-0.994)
3	17	0.963 (0.955-0.971)	0.900 (0.892-0.908)	0.884 (0.839-0.921)	0.899 (0.891-0.907)	0.309 (0.289-0.329)	0.994 (0.991-0.996)
4	11	0.942 (0.931-0.953)	0.901 (0.892-0.909)	0.810 (0.756-0.860)	0.896 (0.888-0.904)	0.291 (0.270-0.313)	0.989 (0.987-0.992)

^a^AUROC: area under the receiver operating characteristic curve.

^b^PPV: positive predictive value.

^c^NPV: negative predictive value.

The AUROCs of Models 1 and 2 for the held-out cohorts were 0.958 (95% CI 0.924-0.991) and 0.943 (95% CI 0.901-0.985), respectively, which were both indifferent from the cross-validation results (*P*=.66 and *P*=.89, respectively). The AUROCs of Models 3 and 4 for the held-out cohorts were 0.949 (95% CI 0.906-0.990) and 0.941 (95% CI 0.903-0.978), respectively, and were also indifferent from the cross-validation results (*P*=.54 and *P*=.95, respectively). The indifferences between the cross-validation and hold-out results revealed that all models had a degree of generalizability to unseen data (Multimedia Appendix 9). Detailed results and the selected variables used at each step of the RFE are presented in Multimedia Appendix 7 and Multimedia Appendix 10.

### Optimal Triage Under Limited Resource Availability

**Figure 3:**
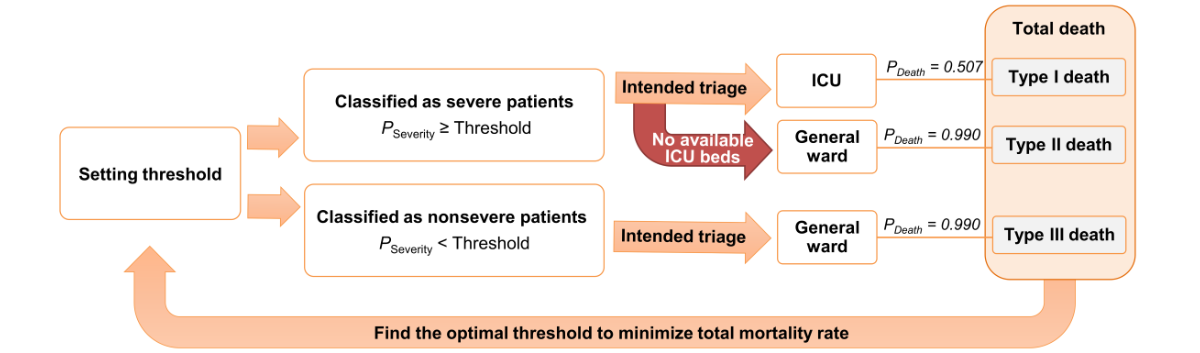
Simulation workflow. Diagram showing how medical resources can be allocated among COVID-19 patients according to the machine learning–based triage system. Patients with a prediction probability exceeding a certain threshold are first triaged to an intensive care unit (ICU) that is currently under its total capacity. Conversely, patients are directed to a general ward if the ICU’s capacity is full or if their severity prediction probability is lower than the threshold. Type I deaths represent those occurring in the ICU. Type II and III deaths represent those of patients who have been directed to the general ward due to ICU unavailability and because they were found to have a disease severity probability lower than the threshold, respectively. We used simulations to obtain the optimal threshold wherein the mortality rate (n [total deaths] / n [total patients] = n [type I death + type II death + type III death] / n [total patients]) is minimized.

The overall DES workflow is illustrated in Figure 3. Mortality rates were minimized at thresholds of 0.1, 0.01, 0.04, and 0.24 for H1, H2, H3, and H4, respectively (Multimedia Appendix 11). The mortality rates showed a convex shape in accordance with these thresholds (Multimedia Appendix 12).

We can infer that as the death rate increases, the threshold should be raised when a large increase is accompanied. While the association between mortality rates and triage thresholds across various patient influx scenarios is inferable through an analysis of historical influx data, it is impractical to draw general conclusions from this information. For example, looking at Multimedia Appendix 11, an upward trend in the optimal threshold and optimized mortality rate occurred when comparing H2, H3, and H4, wherein there was a clear increase in the patient influx volume. However, it is difficult to infer this information when comparing H1 with H3 or H4 because of differences in their multidimensional characteristics, including duration, maximum daily patients, and cumulative patients. To further support our results, we performed additional simulations using patient flow data that were generated using the SIR model with varying R0s.

**Figure 4:**
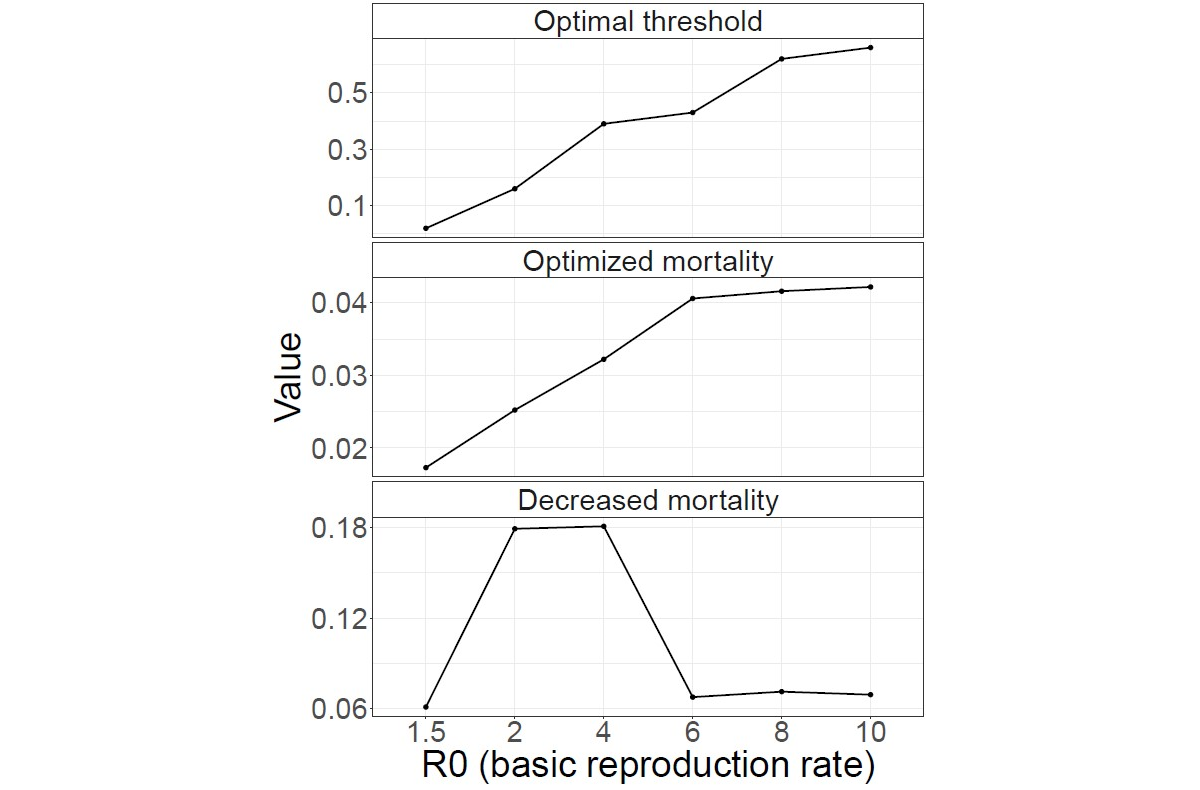
Optimized results of the patient triage simulations for hypothetical influx. Decreased mortality rate = (J-index mortality rate − optimized mortality rate) / J-index mortality rate.

The DES using hypothetical patient influxes revealed that the optimal threshold ranged from 0.02 to 0.66, while the respective minimized mortality rates ranged from 0.017 (1.7%) to 0.042 (4.2%) (Multimedia Appendix 13). The optimal threshold values and minimized mortality rates for each R0 showed that a larger R0 value tends to result in increases in both of these variables. The optimal threshold is increased along with the R0 values to increase precision for severe patients while fully utilizing the ICU. The optimized mortality rates were increased due to an increased proportion of deaths outside the ICU resulting from a larger volume of patient influx. The benefits of utilizing an optimal triage threshold were clear when compared with the conventional Youden Index (J-index) as a benchmark value, which was 0.013. Decreased mortality rates ([J-index mortality rate – optimized mortality rate] / J-index mortality rate) were notably large in a magnitude ranging from 6.1% to 18.1% (Figure 4). Detailed data are provided in Table 4.

**Table 4:** Optimized threshold and its benefits on mortality outcomes according to patient influx settings.

Influx	Optimal threshold	Optimized mortality rate	Decreased mortality rate^a^
H^b^1	0.10	0.022	0.298
H2	0.01	0.015	0.047
H3	0.04	0.019	0.146
H4	0.24	0.031	0.209
R0^c^=1.5	0.02	0.017	0.061
R0=2	0.16	0.025	0.179
R0=4	0.39	0.032	0.181
R0=6	0.43	0.041	0.068
R0=8	0.62	0.042	0.071
R0=10	0.66	0.042	0.069

^a^Decreased mortality rate: (J-index mortality rate – optimized mortality rate) / J-index mortality rate.

^b^H: historical epidemic patient influx scenario.

^c^R0: basic reproduction rate.

**Figure 5:**
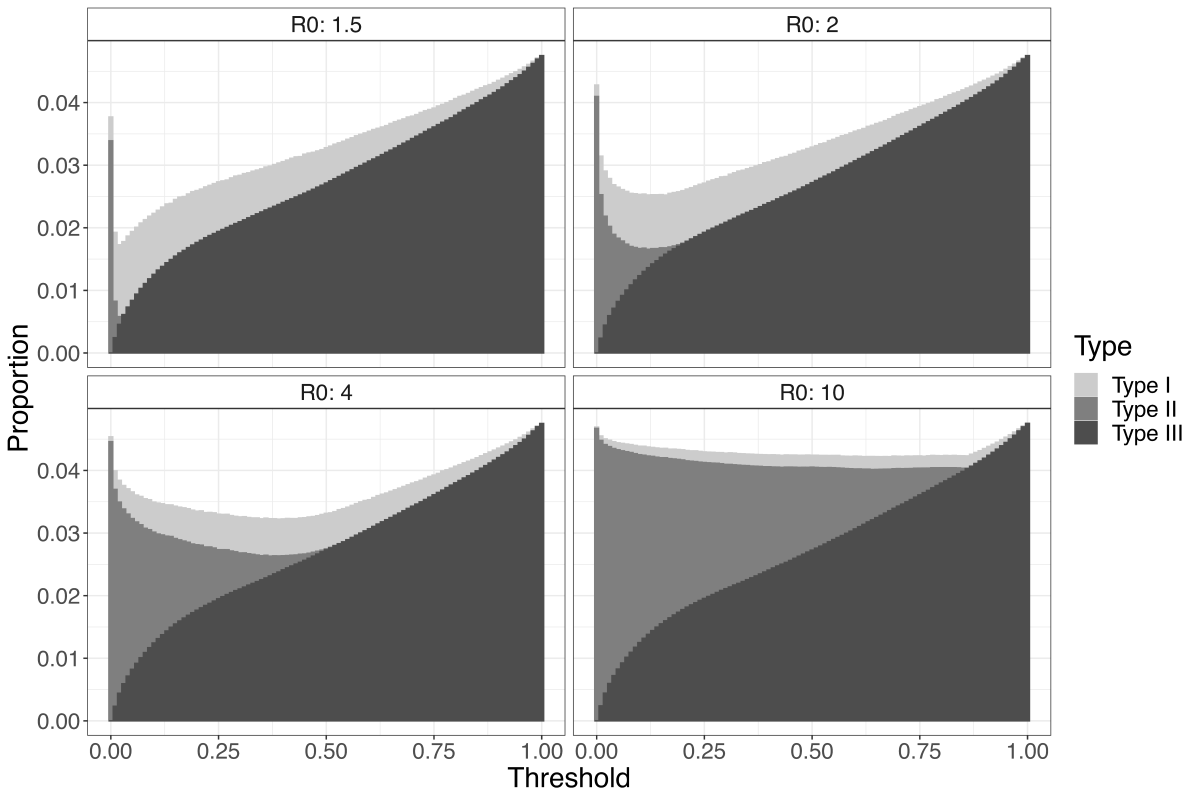
Mortality rates in hypothetical patient influxes are decomposed by death subtype at each threshold. The x-axis represents the threshold, and the y-axis represents the stacked proportion of each death subtype to the total number of patients, calculated as n (death subtype) / n (total patients) at each threshold. R0: basic reproduction rate.

We observed a convex relationship for mortality rates in accordance with the thresholds in Figure 5. The mortality rate was minimized at a point where type I death, which had the lowest *P_death_* (50.7%), was maximized in proportion to total death. For example, when R0 was 1.5, the proportion of type I deaths was maximized at the optimal threshold, accounting for 66.4% of all deaths. However, a threshold that is too low leads to inadequate capacity exhaustion with misclassified nonsevere patients. Consequently, the resulting limited capacity for actual severe patients then decreases the proportion of type I deaths and increases those of type II deaths. Conversely, a threshold that is too high would result in unnecessary rejection for severe patients, which then decreases the proportion of type I deaths and increases those of type III deaths.

In situations of excessively high R0 values and increased ICU demand, increasing the triage threshold to reject more patients will still deplete the ICU capacity. Therefore, adjusting the threshold will mostly result in trade-offs between the numbers of threshold- and capacity-dependent rejections, limiting the influence of threshold adjustment on minimizing patient mortality. In situations of sufficiently low R0 values, the effect of threshold optimization is reduced along with its necessity. Nonetheless, the large reduction in mortality rates among the remaining influxes highlights the substantial benefits of optimizing the patient triage threshold under resource constraints.

### Code Availability

The code used to develop and evaluate this study’s models is available online [21].

## Discussion

### Principal Findings

A distinctive feature of our Model 1 is its high discriminative power with an AUROC that exceeded 0.97 in both cross-validation and hold-out settings. Previous prediction models for determining the clinical deterioration of COVID-19 patients have reported predictive accuracies ranging from 0.77 to 0.91 [2-5]. Additionally, these models require specific diagnostic data, including laboratory data, peripheral oxygen saturation, or radiographic findings, to maintain their predictive accuracies. Moreover, to what extent the performance abilities of these models are maintained during the partial absence of data has not been studied. Given this unmet clinical need, we developed Model 1. In addition, we confirmed that our feature-eliminated models maintained an adequate discriminative power even in the partial absence of data. The advantages of our feature-eliminated models include not only their increased generalizability to unseen data, but also their applicability within scenarios wherein there is limited medical data. We have uploaded Model 3 online to be implemented in clinical practice. Given the acute exacerbation of pneumonia in COVID-19 patients, our model can also be used to re-evaluate hospitalized patients in the short term, so that individuals whose clinical manifestations are likely to worsen can be identified as early as possible [22].

A noteworthy feature of our model is its ability to discriminate between patient-specific factors contributing to disease exacerbation and their individual contributions using SHAP values. Current COVID-19 treatment guidelines provide recommendations based on the average-risk patient under limited available insights into their disease stage [10]. These recommendations provide a one-size-fits-all approach to all patients, which is problematic for those with more complex or atypical disease presentations. Our model obviates the need for arbitrary patient risk groupings and is therefore useful in maximizing survival odds based on individual risk stratification. Furthermore, our model can be integrated into electronic medical record systems, which utilize coding algorithms, as a notification system that helps in the early identification of disease exacerbation risk factors.

The validity of our model is supported by the high consistency between the results of its interpretation using SHAP and previously reported prognosticators of COVID-19 severity [23-28]. We noted that old age, followed by lymphopenia and thrombocytopenia, exhibited the highest Shapley values for disease exacerbation. We presume that age interacts with relevant features in older adults, including poor functional performance and increased frailty, which are associated with adverse outcomes and increased mortality among patients with respiratory syndromes [29]. Our findings also support literature indicating that lymphopenia plays an important role in COVID-19 exacerbation [25-28]. Lymphopenia is characterized by the lowering of lymphocytes due to injured alveolar epithelial cells and is commonly observed in COVID-19 patients [30]. Consistent with previous studies, thrombocytopenia was also found to be associated with adverse COVID-19 outcomes [26,31]. It has been suggested that a reduction or morphological alternation in the pulmonary capillary bed exerts pathological platelet defragmentation because the lungs are a platelet release site with mature megakaryocytes [32]. Our prediction model supports the notion that the early identification of COVID-19 infection, before a hematological crisis occurs, is necessary for ensuring a better prognosis.

There is no existing study that has examined COVID-19 severity prediction models in an attempt to provide an explicit solution for the delivery of optimal triage using threshold modification that accounts for limited resource availability. We employed DES in our Model 3 to examine discrimination thresholds that are usable in an adaptive manner across various patient influx scenarios and the related health care resource availability. Our simulations revealed that applying the optimal thresholds of both historical and generated patient influxes will minimize the mortality rate of each patient influx scenario. Our hypothesis is supported by the significant differences found in mortality rates between the J-index and our optimized thresholds when applied to the expected patient influx volumes. This observation supports the potential usability of our model to substantially reduce COVID-19 mortality rates through the appropriate and effective adjustment of triage thresholds.

### Limitations

One limitation of our study is its incorporation of a single national cohort of Asian ethnicity with a relatively small sample size, which impacts the generalizability of our findings. External validation using a more multiethnic population is thus needed to determine if a similar discrimination performance occurs among other ethnic groups. However, to ensure our model’s robustness, we implemented 10-fold cross-validation with additional confirmation using a hold-out cohort. Second, the triage threshold was evaluated using a simulation. Simulations do not yield concrete answers and are unable to assess all kinds of potential situations [33]. Third, the applicability of utilizing SHAP values to discriminate patient-specific contributing factors for disease exacerbation has not been prospectively validated. Whether the early identification of disease exacerbation risk factors and their individual contributions can result in a better prognosis would need to be validated after the implementation of our online system into clinical practice. Lastly, clinical data, including self-reported measurements, may not be objectively interpreted, and models utilizing these parameters should be interpreted cautiously.

### Conclusions

We developed and validated a robust prediction model, with an explanatory feature, that offers an effective means of enhancing the efficiency of COVID-19 triage. We further proposed an adaptive triage model that utilizes both patient influx volume and the capacity of a health care system to minimize mortality rates within the scope of resource limits. Our model has the potential for effective application because it is available online for patients and providers in both inpatient and outpatient settings. Overall, our results imply that COVID-19 treatment plans need to integrate both medical and health care management expertise to guarantee maximum efficacy.

## Appendix

Multimedia Appendix 1 Epidemic incidence curves of historical patient influxes of COVID-19 in South Korea.

Multimedia Appendix 2 Susceptible-infectious-recovered (SIR) simulated patient influx.

Multimedia Appendix 3 Prediction probability distribution graph of patients in the out-of-fold samples.

Multimedia Appendix 4 Performance of the models according to the World Health Organization Ordinal Scale for Clinical Improvement.

Multimedia Appendix 5 Patient-specific Shapley additive explanations (SHAP) plots.

Multimedia Appendix 6 Changes to the model’s performance after applying recursive feature elimination (RFE) (Model 1).

Multimedia Appendix 7 Areas under the receiver operating characteristic curve (AUROCs) at each step of the recursive feature elimination (RFE) and <italic>P</italic> values of differences in AUROC values for Models 1 and 2.

Multimedia Appendix 8 Changes to the model’s performance after applying recursive feature elimination (RFE) (Model 2).

Multimedia Appendix 9 Performance of the models in the hold-out cohort.

Multimedia Appendix 10 Order of feature importance for each model.

Multimedia Appendix 11 Optimized results of the patient triage simulations for the historical influx.

Multimedia Appendix 12 Mortality rates of the historical patient influx scenarios according to each threshold and at each threshold across different scenarios.

Multimedia Appendix 13 Mortality rates of the hypothetical patient influx scenarios according to each threshold and at each threshold across different scenarios.

## Abbreviations

AUROC: area under the receiver operating characteristic curve
DES: discrete-event simulation
ICU: intensive care unit
KDCA: Korea Disease Control and Prevention Agency
LR: logistic regression
NPV: negative predictive value
OSCI: Ordinal Scale for Clinical Improvement
PPV: positive predictive value
R0: basic reproduction rate
RFE: recursive feature elimination
SHAP: Shapley additive explanations
SIR: susceptible-infectious-recovered
WHO: World Health Organization
XGBoost: extreme gradient boosting
