# Supplementary material for: Optimal Triage for COVID-19 Patients Under Limited Health Care Resources With a Parsimonious Machine Learning Prediction Model and Threshold Optimization Using Discrete-Event Simulation: Development Study
Source: JMIR Med Inform. 2021 Nov 2;9(11):e32726. doi: 10.2196/32726 (PMC8565604; doi:10.2196/32726)
Supplement: Multimedia Appendix 1 [file medinform_v9i11e32726_app1.docx]

**Multimedia Appendix 1.** Epidemic incidence curves of historical patient influxes of COVID-19 in South Korea.


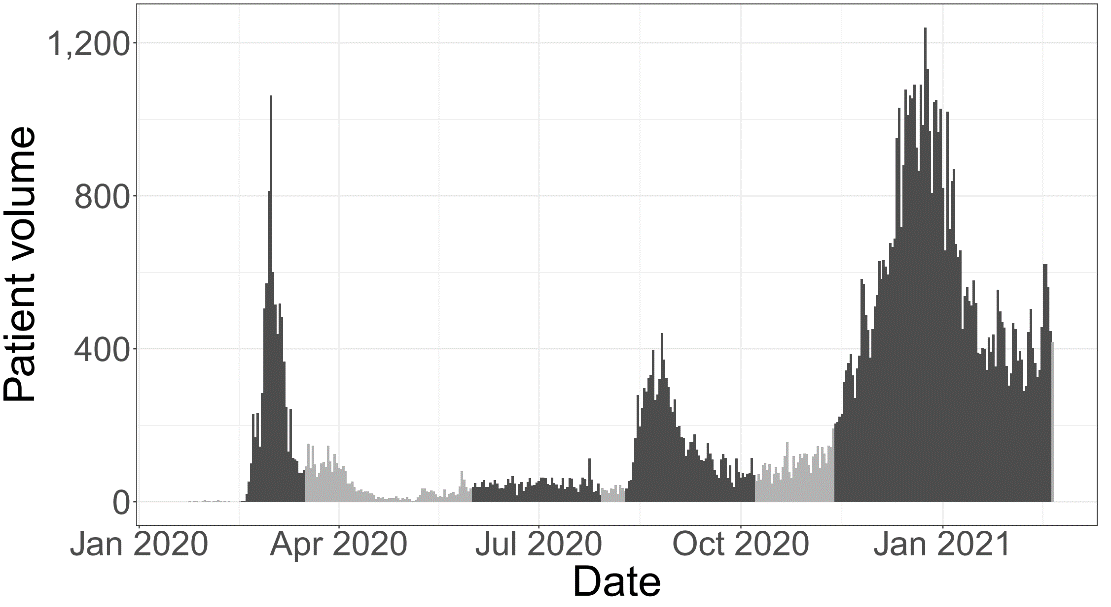


|  | **H 1** | **H 2** | **H 3** | **H 4** |
| --- | --- | --- | --- | --- |
| **Period** | 17^th^ Feb - 17^th^ Mar | 1^st^ Jun - 30^th^ Jul | 10^th^ Aug - 8^th^ Oct | 13^th^ Nov - 20^th^ Feb |
| **Duration (days)** | 30 | 60 | 60 | 100 |
| **Cumulative patients** | 8,384 | 2,802 | 9,850 | 58,859 |
| **Maximum daily patients**  **(= maximum height)** | 1,062 | 113 | 441 | 1,240 |
