# Supplementary material for: Optimal Triage for COVID-19 Patients Under Limited Health Care Resources With a Parsimonious Machine Learning Prediction Model and Threshold Optimization Using Discrete-Event Simulation: Development Study
Source: JMIR Med Inform. 2021 Nov 2;9(11):e32726. doi: 10.2196/32726 (PMC8565604; doi:10.2196/32726)
Supplement: Multimedia Appendix 2 [file medinform_v9i11e32726_app2.docx]

**Multimedia Appendix 2.** Susceptible-infectious-recovered (SIR) simulated patient influx.


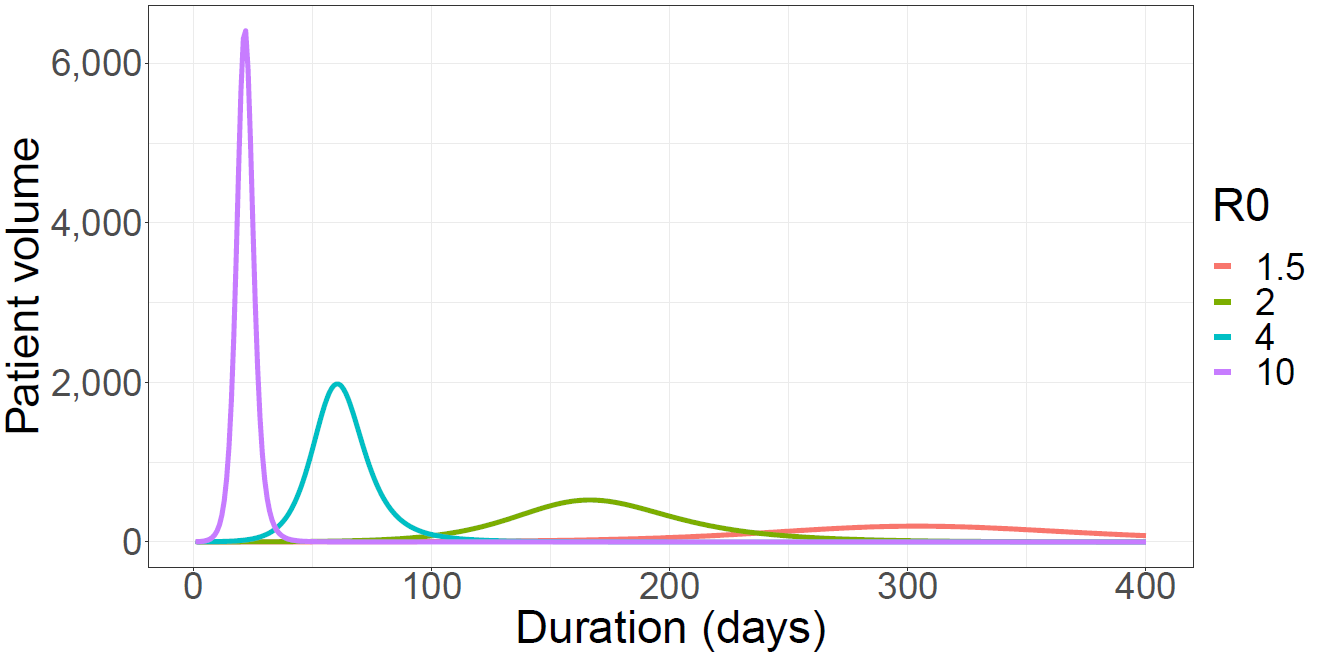


| **R0** | **1.5** | **2** | **4** | **10** |
| --- | --- | --- | --- | --- |
| **Duration (days)** | 677 | 416 | 198 | 65 |
| **Cumulative patients** | 34,965 | 47,802 | 58,799 | 59,993 |
| **Maximum daily patients**  **(= maximum height)** | 198 | 526 | 1,980 | 6,407 |

R0 = basic reproduction rate.
