# Supplementary material for: Optimal Triage for COVID-19 Patients Under Limited Health Care Resources With a Parsimonious Machine Learning Prediction Model and Threshold Optimization Using Discrete-Event Simulation: Development Study
Source: JMIR Med Inform. 2021 Nov 2;9(11):e32726. doi: 10.2196/32726 (PMC8565604; doi:10.2196/32726)

**Multimedia Appendix 3.** Prediction probability distribution graph of patients in the out-of-fold samples.


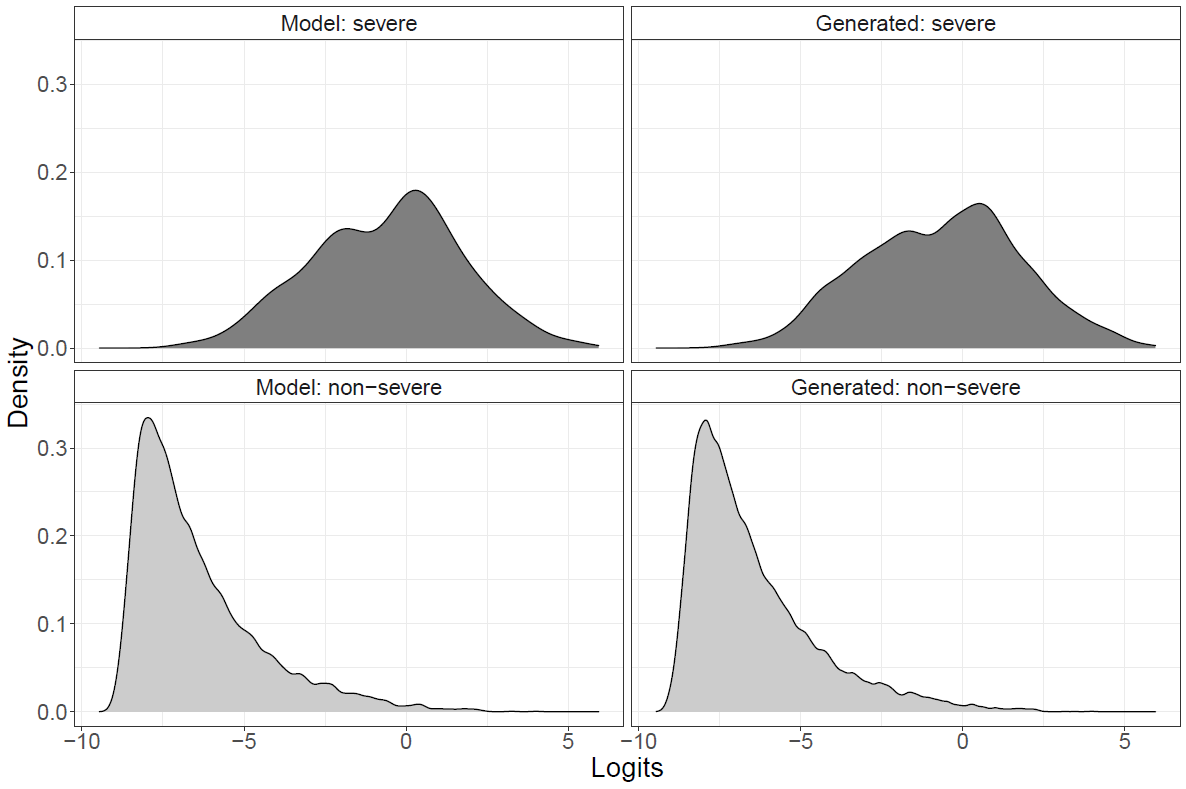

Supplement: Multimedia Appendix 3 [file medinform_v9i11e32726_app3.docx]
