# Supplementary material for: Optimal Triage for COVID-19 Patients Under Limited Health Care Resources With a Parsimonious Machine Learning Prediction Model and Threshold Optimization Using Discrete-Event Simulation: Development Study
Source: JMIR Med Inform. 2021 Nov 2;9(11):e32726. doi: 10.2196/32726 (PMC8565604; doi:10.2196/32726)
Supplement: Multimedia Appendix 4 [file medinform_v9i11e32726_app4.docx]

**Multimedia Appendix 4.** Performance of the models according to the World Health Organization Ordinal Scale for Clinical Improvement.

| **WHO OSCI** | **Model** | **AUROC** | **Specificity** | **Sensitivity** | **Accuracy** | **PPV** | **NPV** | ***P*** |
| --- | --- | --- | --- | --- | --- | --- | --- | --- |
| ≥ 5 | LR | 0.915  (0.882 - 0.941) | 0.901  (0.891 - 0.908) | 0.766  (0.695 - 0.830) | 0.893  (0.882 - 0.905) | 0.300  (0.270 - 0.330) | 0.986  (0.982 - 0.990) | *0.030* |
|  | XGB | 0.950 (0.936 - 0.961) | 0.900  (0.891 - 0.908) | 0.882  (0.838 - 0.919) | 0.899 (0.890 - 0.907) | 0.333 (0.312 - 0.356) | 0.993  (0.990 - 0.995) |  |
| ≥ 6 | LR | 0.938  (0.911 - 0.959) | 0.900  (0.888 - 0.912) | 0.814  (0.737 - 0.881) | 0.896  (0.885 - 0.907) | 0.273 (0.246 - 0.302) | 0.991  (0.987 - 0.994) | *0.039* |
|  | XGB | 0.965  (0.958 - 0.972) | 0.900  (0.892 - 0.908) | 0.905  (0.868 - 0.942) | 0.901  (0.892 - 0.909) | 0.314  (0.295 - 0.335) | 0.995  (0.993 - 0.997) |  |
| ≥ 7 | LR | 0.938  (0.903 - 0.963) | 0.900  (0.888 - 0.912) | 0.857  (0.790 - 0.924) | 0.898  (0.887 - 0.910) | 0.259  (0.234 - 0.289) | 0.994  (0.993 - 0.997) | *0.060* |
|  | XGB | 0.966  (0.959 - 0.973) | 0.900  (0.892 - 0.909) | 0.902  (0.862 - 0.938) | 0.900  (0.892 - 0.909) | 0.297  (0.278 - 0.318) | 0.995  (0.993 - 0.997) |  |
| ≥ 8 | LR | 0.940  (0.907 - 0.964) | 0.900  (0.889 - 0.911) | 0.867  (0.796 - 0.929) | 0.899  (0.887 - 0.909) | 0.248  (0.223 - 0.273) | 0.994  (0.991 - 0.997) | *0.062* |
|  | XGB | 0.968  (0.961 - 0.975) | 0.900  (0.892 - 0.908) | 0.903  (0.861 - 0.940) | 0.900  (0.892 - 0.908) | 0.288  (0.270 - 0.307) | 0.995  (0.993 - 0.997) |  |

AUROC = area under the receiver operating characteristic, CSS = clinical severity score, LR = logistic regression, NPV = negative predictive value, PPV = positive predictive value, WHO OSCI = World Health Organization Ordinal Scale for Clinical Improvement, XGB = extreme gradient boosting.
