# Supplementary material for: Optimal Triage for COVID-19 Patients Under Limited Health Care Resources With a Parsimonious Machine Learning Prediction Model and Threshold Optimization Using Discrete-Event Simulation: Development Study
Source: JMIR Med Inform. 2021 Nov 2;9(11):e32726. doi: 10.2196/32726 (PMC8565604; doi:10.2196/32726)

**Multimedia Appendix 5.** Patient-specific Shapley additive explanations (SHAP) plots.

The top 10 features, which were ranked for each patient by averaging their SHAP values, depicting the model’s predictive behaviors. In this specific example, the SHAP plot for an actual severe patient shows that their age, lymphocyte count, and experiencing shortness of breath significantly affected their prediction output in a positive direction. However, their white blood cell count and hematocrit, as well as their hemoglobin and BMI, contributed in a negative direction. On the other hand, the SHAP plot for an actual non-severe patient shows that their age, lymphocyte count, platelet counts, and hemoglobin significantly affected their prediction output in the negative direction.


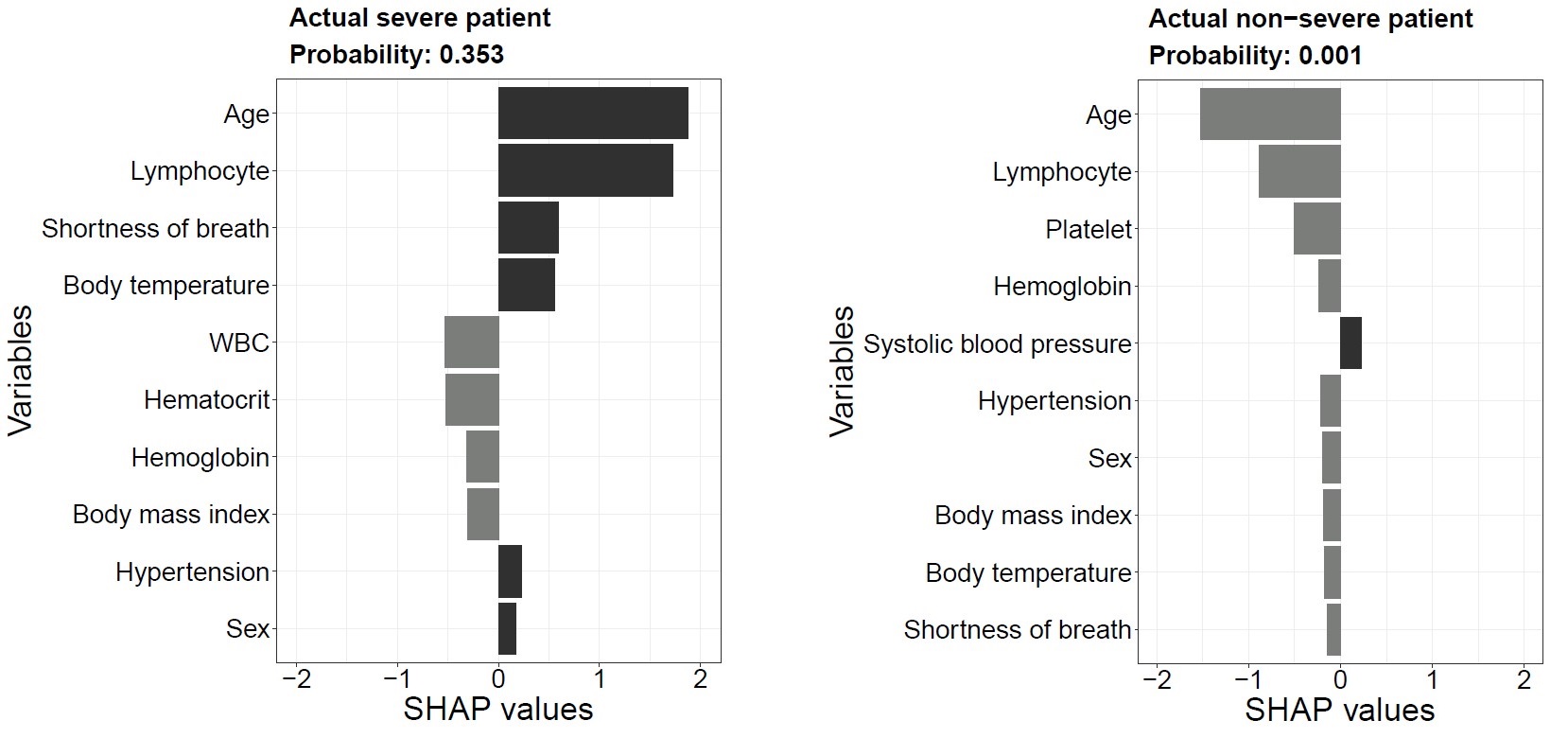

Supplement: Multimedia Appendix 5 [file medinform_v9i11e32726_app5.docx]
