# Supplementary material for: Optimal Triage for COVID-19 Patients Under Limited Health Care Resources With a Parsimonious Machine Learning Prediction Model and Threshold Optimization Using Discrete-Event Simulation: Development Study
Source: JMIR Med Inform. 2021 Nov 2;9(11):e32726. doi: 10.2196/32726 (PMC8565604; doi:10.2196/32726)

**Multimedia Appendix 6.** Changes to the model’s performance after applying recursive feature elimination (RFE) (Model 1).

The outcome of the entire RFE is presented in terms of its AUROC values. RFE was performed on Model 1, which consisted of 37 variables, including five laboratory variables. Model 1 during the first iteration was developed using 37 variables, while Model 3 was developed using 17 variables. In Model 1, pregnancy and pregnancy week variables showed SHAP values of 0 and were eliminated at the first iteration.


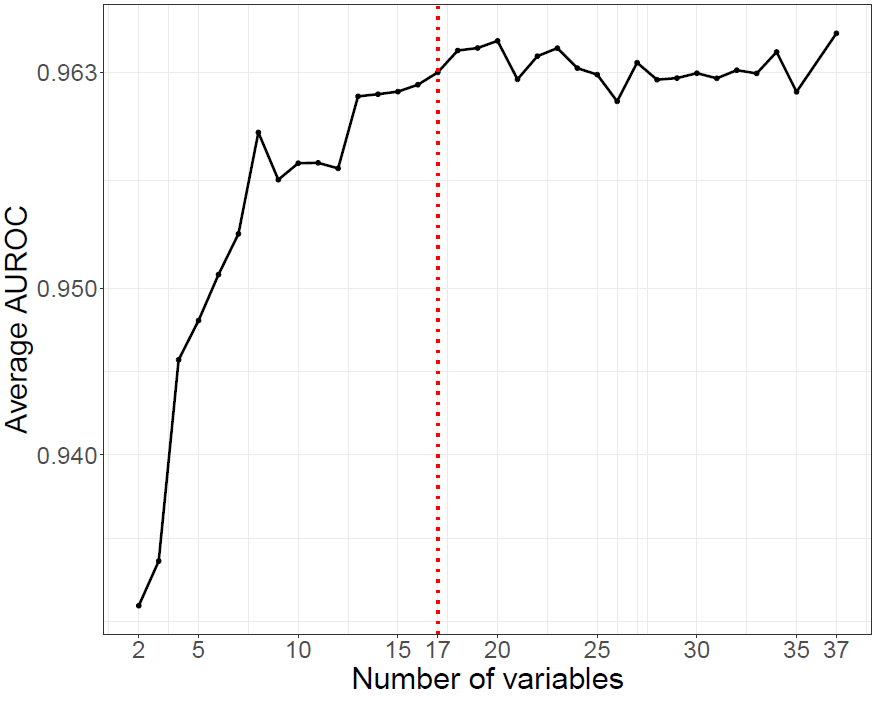

Supplement: Multimedia Appendix 6 [file medinform_v9i11e32726_app6.docx]
