# Supplementary material for: Optimal Triage for COVID-19 Patients Under Limited Health Care Resources With a Parsimonious Machine Learning Prediction Model and Threshold Optimization Using Discrete-Event Simulation: Development Study
Source: JMIR Med Inform. 2021 Nov 2;9(11):e32726. doi: 10.2196/32726 (PMC8565604; doi:10.2196/32726)
Supplement: Multimedia Appendix 7 [file medinform_v9i11e32726_app7.docx]

**Multimedia Appendix 7.** Areas under the receiver operating characteristic curve (AUROCs) at each step of the recursive feature elimination (RFE) and *P* values of differences in AUROC values for Models 1 and 2.

| **Number of variables** | **Model 1** | | **Model 2** | |
| --- | --- | --- | --- | --- |
|  | **AUROC** | ***P*** | **AUROC** | ***P*** |
| 1 | 0.889 | *0.000* | 0.889 | *0.000* |
| 2 | 0.931 | *0.000* | 0.917 | *0.000* |
| 3 | 0.934 | *0.000* | 0.921 | *0.000* |
| 4 | 0.946 | *0.000* | 0.932 | *0.001* |
| 5 | 0.948 | *0.000* | 0.936 | *0.008* |
| 6 | 0.951 | *0.000* | 0.937 | *0.009* |
| 7 | 0.953 | *0.000* | 0.934 | *0.001* |
| 8 | 0.959 | *0.003* | 0.936 | *0.007* |
| 9 | 0.956 | *0.000* | 0.935 | *0.000* |
| 10 | 0.957 | *0.000* | 0.938 | *0.007* |
| 11 | 0.958 | *0.000* | 0.942 | *0.137* |
| 12 | 0.957 | *0.000* | 0.941 | *0.043* |
| 13 | 0.961 | *0.018* | 0.939 | *0.014* |
| 14 | 0.962 | *0.018* | 0.942 | *0.087* |
| 15 | 0.962 | *0.015* | 0.942 | *0.063* |
| 16 | 0.962 | *0.021* | 0.941 | *0.015* |
| 17 | 0.963 | *0.080* | 0.942 | *0.108* |
| 18 | 0.964 | *0.252* | 0.943 | *0.051* |
| 19 | 0.964 | *0.379* | 0.945 | *0.435* |
| 20 | 0.965 | *0.392* | 0.943 | *0.024* |
| 21 | 0.963 | *0.016* | 0.946 | *0.556* |
| 22 | 0.964 | *0.352* | 0.946 | *0.640* |
| 23 | 0.964 | *0.510* | 0.948 | *0.551* |
| 24 | 0.963 | *0.107* | 0.947 | *0.988* |
| 25 | 0.963 | *0.077* | 0.945 | *0.517* |
| 26 | 0.961 | *0.002* | 0.946 | *0.693* |
| 27 | 0.964 | *0.141* | 0.946 | *0.305* |
| 28 | 0.962 | *0.014* | 0.945 | *0.437* |
| 29 | 0.963 | *0.027* | 0.947 | *0.672* |
| 30 | 0.963 | *0.043* | 0.942 | *0.004* |
| 31 | 0.963 | *0.116* | 0.946 | *0.686* |
| 32 | 0.963 | *0.054* | 0.946 | *-* |
| 33 | 0.963 | *0.173* | - | *-* |
| 34 | 0.964 | *0.378* | - | *-* |
| 35 | 0.962 | *0.009* | - | *-* |
| 37 | 0.965 | *-* | - | *-* |

For Model 1, pregnancy and pregnancy week variables showed SHAP values of 0 and were eliminated at the beginning of the RFE. As Model 3 was derived from Model 1 by RFE, Model 3 corresponded to the model with the top 17 variables in the Model 1 column (i.e., 20 variables were removed from Model 1). Likewise, Model 4 corresponded to the model with the top 11 variables in the Model 2 column.
