# Supplementary material for: Optimal Triage for COVID-19 Patients Under Limited Health Care Resources With a Parsimonious Machine Learning Prediction Model and Threshold Optimization Using Discrete-Event Simulation: Development Study
Source: JMIR Med Inform. 2021 Nov 2;9(11):e32726. doi: 10.2196/32726 (PMC8565604; doi:10.2196/32726)

**Multimedia Appendix 8.** Changes to the model’s performance after applying recursive feature elimination (RFE) (Model 2).

The outcome of the entire RFE is presented in terms of its AUROC values. RFE was performed on Model 2, which consisted of 32 variables, in which five laboratory variables were excluded. Model 2 at the first iteration was developed based on 32 variables, while Model 4 was developed using 11 variables.


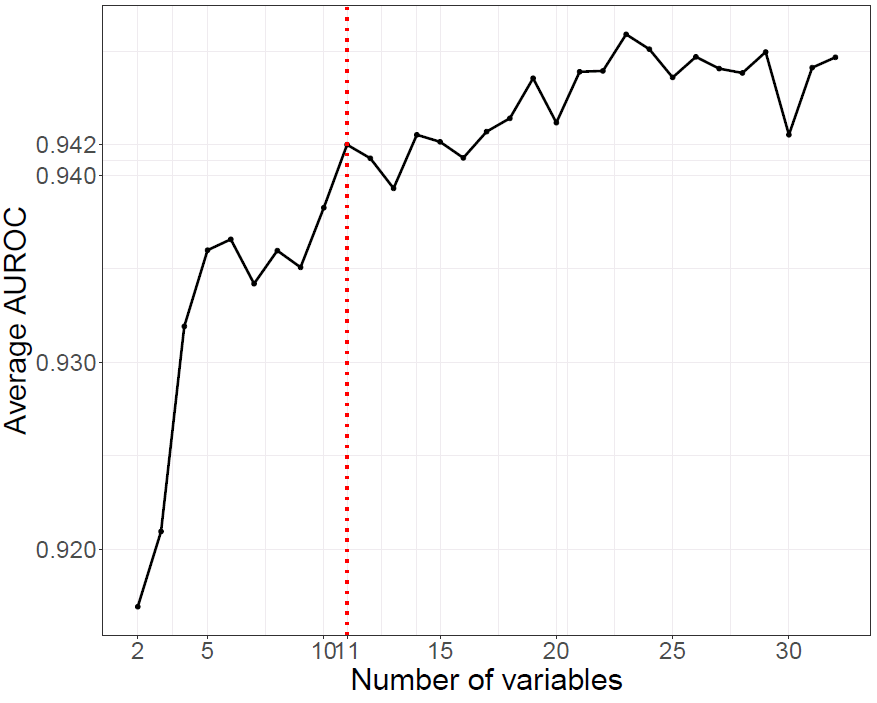

Supplement: Multimedia Appendix 8 [file medinform_v9i11e32726_app8.docx]
