# Supplementary material for: Optimal Triage for COVID-19 Patients Under Limited Health Care Resources With a Parsimonious Machine Learning Prediction Model and Threshold Optimization Using Discrete-Event Simulation: Development Study
Source: JMIR Med Inform. 2021 Nov 2;9(11):e32726. doi: 10.2196/32726 (PMC8565604; doi:10.2196/32726)
Supplement: Multimedia Appendix 9 [file medinform_v9i11e32726_app9.docx]

**Multimedia Appendix 9.** Performance of the models in the hold-out cohort.

| **Model** | **Number of variables** | **AUROC** | **Specificity** | **Sensitivity** | **Accuracy** | **PPV** | **NPV** | ***P*** |
| --- | --- | --- | --- | --- | --- | --- | --- | --- |
| 1 | 37 | 0.958  (0.924-0.991) | 0.882  (0.854-0.908) | 0.966  (0.897-1.000) | 0.887  (0.860-0.911) | 0.309  (0.262-0.367) | 0.998  (0.994-1.000) | *0.663* |
| 2 | 32 | 0.943  (0.901-0.985) | 0.907  (0.882-0.931) | 0.828  (0.690-0.966) | 0.902  (0.878-0.926) | 0.325  (0.265-0.400) | 0.990  (0.982-0.998) | *0.889* |
| 3 | 17 | 0.949  (0.906-0.99) | 0.888  (0.862-0.914) | 0.966  (0.897-1.000) | 0.892  (0.867-0.917) | 0.319  (0.270-0.378) | 0.998 (0.994-1.000) | *0.541* |
| 4 | 11 | 0.941  (0.903-0.978) | 0.912  (0.890-0.935) | 0.828  (0.690-0.931) | 0.908  (0.885-0.931) | 0.338  (0.274-0.415) | 0.990  (0.982-0.996) | *0.950* |

AUROC = area under the receiver operating characteristic curve, NPV = negative predictive value, PPV = positive predictive value.
