# Supplementary material for: Optimal Triage for COVID-19 Patients Under Limited Health Care Resources With a Parsimonious Machine Learning Prediction Model and Threshold Optimization Using Discrete-Event Simulation: Development Study
Source: JMIR Med Inform. 2021 Nov 2;9(11):e32726. doi: 10.2196/32726 (PMC8565604; doi:10.2196/32726)
Supplement: Multimedia Appendix 10 [file medinform_v9i11e32726_app10.docx]

**Multimedia Appendix 10.** Order of feature importance for each model.

| **Order** | **Model 1** | **Model 2** | **Model 3** | **Model 4** |
| --- | --- | --- | --- | --- |
| 1 | Age | Age | Age | Age |
| 2 | Lymphocyte | Shortness of breath | Lymphocyte | Shortness of breath |
| 3 | Platelet | Body mass index | Platelet | Body mass index |
| 4 | Body mass index | Sex | Body mass index | Sex |
| 5 | Hematocrit | Body temperature | Hematocrit | Body temperature |
| 6 | Shortness of breath | Heart rate | Shortness of breath | Heart rate |
| 7 | Sex | Hypertension | Sex | Hypertension |
| 8 | Body temperature | Systolic blood pressure | Body temperature | Systolic blood pressure |
| 9 | Heart rate | Diabetes mellitus | Heart rate | Diabetes mellitus |
| 10 | White blood cell | Dementia | White blood cell | Dementia |
| 11 | Hemoglobin | Diastolic blood pressure | Hemoglobin | Diastolic blood pressure |
| 12 | Hypertension | Rhinorrhea | Hypertension | N/A |
| 13 | Dementia | Fever | Dementia | N/A |
| 14 | Diabetes mellitus | Cough | Diabetes mellitus | N/A |
| 15 | Systolic blood pressure | Headache | Systolic blood pressure | N/A |
| 16 | Diastolic blood pressure | Sore throat | Diastolic blood pressure | N/A |
| 17 | Altered consciousness | Altered consciousness | Altered consciousness | N/A |
| 18 | Rhinorrhea | Malignancy | N/A | N/A |
| 19 | Headache | Sputum | N/A | N/A |
| 20 | Cough | Myalgia | N/A | N/A |
| 21 | Fatigue | Cardiovascular disease | N/A | N/A |
| 22 | Cardiovascular disease | Chronic kidney disease | N/A | N/A |
| 23 | Malignancy | Diarrhea | N/A | N/A |
| 24 | Myalgia | Vomiting | N/A | N/A |
| 25 | Sputum | Chronic liver disease | N/A | N/A |
| 26 | Fever | Fatigue | N/A | N/A |
| 27 | Chronic kidney disease | Asthma | N/A | N/A |
| 28 | Chronic liver disease | Heart failure | N/A | N/A |
| 29 | Heart failure | Chronic obstructive pulmonary disease | N/A | N/A |
| 30 | Diarrhea | Autoimmune disease | N/A | N/A |
| 31 | Autoimmune disease | Pregnancy | N/A | N/A |
| 32 | Vomiting | Pregnancy Weeks | N/A | N/A |
| 33 | Asthma | N/A | N/A | N/A |
| 34 | Sore throat | N/A | N/A | N/A |
| 35 | Chronic obstructive pulmonary disease | N/A | N/A | N/A |
| 37 | Pregnancy | N/A | N/A | N/A |
| 37 | Pregnancy Weeks | N/A | N/A | N/A |
