# Supplementary material for: Optimal Triage for COVID-19 Patients Under Limited Health Care Resources With a Parsimonious Machine Learning Prediction Model and Threshold Optimization Using Discrete-Event Simulation: Development Study
Source: JMIR Med Inform. 2021 Nov 2;9(11):e32726. doi: 10.2196/32726 (PMC8565604; doi:10.2196/32726)

**Multimedia Appendix 11.** Optimized results of the patient triage simulations for the historical influx.

Decreased mortality rate = (J index mortality rate - optimized mortality rate) / J index mortality rate.


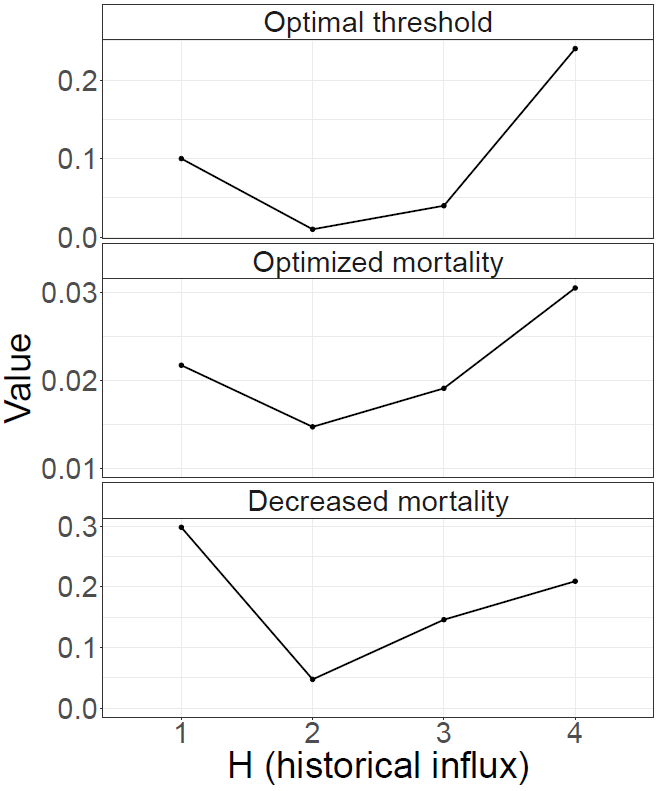

Supplement: Multimedia Appendix 11 [file medinform_v9i11e32726_app11.docx]
