# Supplementary material for: Optimal Triage for COVID-19 Patients Under Limited Health Care Resources With a Parsimonious Machine Learning Prediction Model and Threshold Optimization Using Discrete-Event Simulation: Development Study
Source: JMIR Med Inform. 2021 Nov 2;9(11):e32726. doi: 10.2196/32726 (PMC8565604; doi:10.2196/32726)

**Multimedia Appendix 13.** Mortality rates of the hypothetical patient influx scenarios according to each threshold and at each threshold across different scenarios.

Black dots represent the optimized mortality rate and the respective optimal threshold for each patient influx scenario.


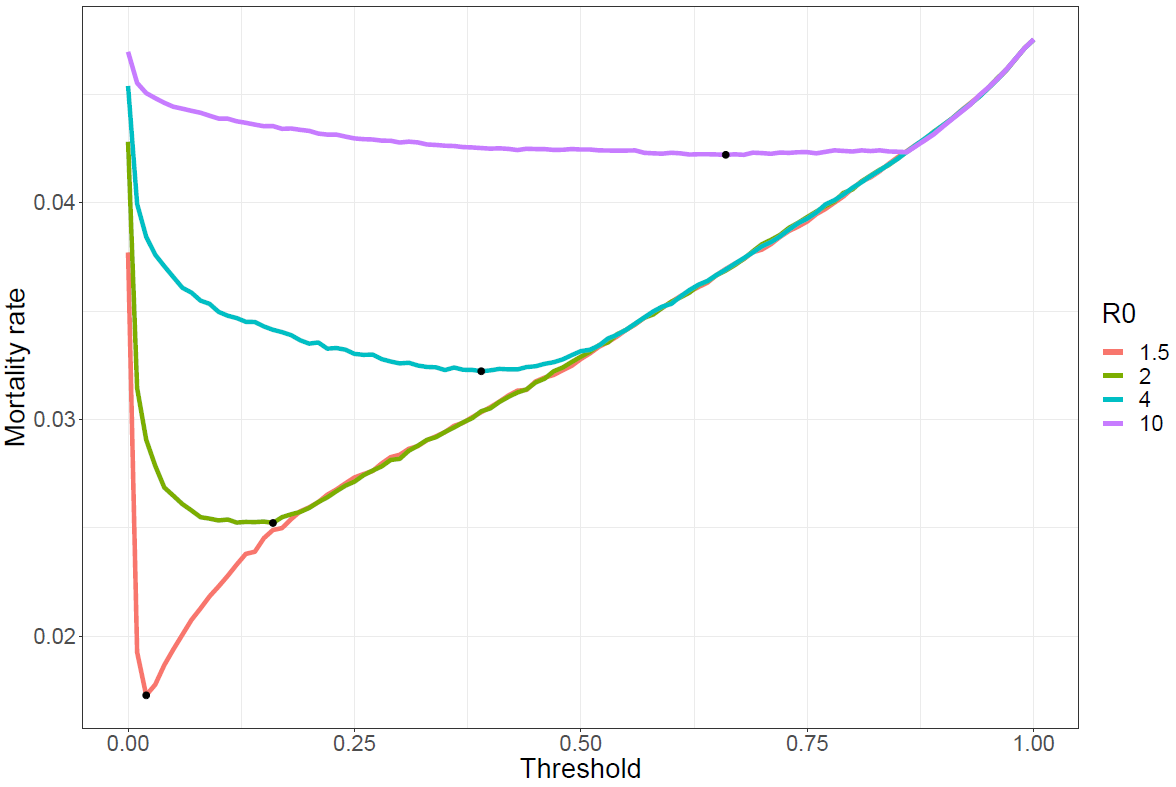

Supplement: Multimedia Appendix 13 [file medinform_v9i11e32726_app13.docx]
